# Goat Milk‐Based Infant Formula and the Prevalence of Gastrointestinal Symptoms in Infants: A Real‐World‐Evidence Study From Brazil, Mexico, Russia, and the Netherlands

**DOI:** 10.1002/hsr2.70448

**Published:** 2025-02-10

**Authors:** Karen Knipping, Juliane Böhme, Dominique Goossens, Linde van Lee, Lucie van der Zee

**Affiliations:** ^1^ Ausnutria B.V. Zwolle The Netherlands; ^2^ Department of Human Nutrition Wageningen University Wageningen The Netherlands

**Keywords:** consumer survey, cow's milk infant formula, CoMiSS, gastrointestinal symptoms, infantsgoat‐milk infant formula

## Abstract

**Background and Aims:**

Infants often experience gastro‐intestinal (GI) symptoms whereby their nutrition might play a role in the occurrence of these symptoms. Evidence suggests that consuming goat milk‐based infant formula (GMF) may provide relief. Therefore, a study was performed to assess the prevalence of GI symptoms in infants and the role of GMF.

**Methods:**

From June 2021 until November 2022, a cross‐sectional observational survey was conducted in Brazil, Mexico, Russia, and The Netherlands by recruiting parents/legal guardians through social media. GI symptoms after introduction of GMF were questioned using the validated Cow's Milk‐Related Symptom Score (CoMiSS; scoring 0–33), lower scores indicate fewer symptoms. Occurrence of crying, gassiness, eczema, respiratory symptoms and stool consistency were asked retrospectively before and ≥ 14 days after introduction of GMF.

**Results:**

The study population (*n* = 425) of GMF‐consumers mostly consisted of 0‐6 months‐old infants with a median age of 5 months. Overall, CoMiSS was low among GMF‐consumers (median 1.00, IQR 4.00, range 0–14) of which 89% had CoMiSS ≤ 6 (*n* = 317), indicating (severity of) symptoms commonly present in healthy infants. After introduction of GMF, the majority of infants had lower symptom scores of gassiness (87%, *n* = 128), skin symptoms (78%, *n* = 32), watery stools/diarrhea (80%, *n* = 32) or hard stools/constipation (84%, *n* = 57), and crying (87%, *n* = 122). The CoMiSS in GMF‐consumers was significantly lower, meaning fewer symptoms, compared to GMF‐non‐consumers (*p* = < 0.001). CoMiSS was similar among categories of age, sex, and country of residence.

**Conclusion:**

This sample of infants fed GMF showed a low prevalence of GI symptoms and parents reported lower prevalence of GI symptoms after introduction of GMF based on retrospectively questionnaires. CoMiSS in GMF‐consumers was significantly lower when compared to GMF‐non‐consumers. These results give indications of a potential benefit of GMF in managing GI symptoms in infants. Clinicaltrial. gov identifier NCT06755424.

## 1 Introduction

Adequate early life nutrition is essential for optimal health and growth of infants as well as for their health later in life [1, 2]. Exclusive breastfeeding in the first 6 months of life is considered as the most optimal source of nutrition for infants. Despite the World Health Organization (WHO) recommendations, more than 50% of infants globally are not exclusively breastfed during their first 6 months of life, and are therefore dependent on infant formula for their nutrition from early infancy [3]. Over the years, a variety of infant formulas have been developed; mostly based on cow's milk, whereas infant formula based on goat milk has been introduced in the last decades. The tolerability and nutritional adequacy of GMF has been previously examined in several studies, showing that GMF provides adequate nutrition for growth and is safe and suitable to use for infants [4]. In 2012, the European Food Safety Authority (EFSA) stated that goat milk protein is suitable as a protein source in infant formulas [5], in 2017 GRAS (generally recognized as safe) notification for nonfat dry goats' milk and goat whey protein concentrate in infant formula [6] was granted by the Food and Drug Administration (FDA).

Gastrointestinal (GI) issues such as regurgitation, watery stools, constipation; skin issues like eczema and urticaria; respiratory issues such as wheezing; and general symptoms such as poor growth and infantile colic occur in 15%–20% of infants [7] and these symptoms can be related to cow's milk protein intake. The CoMiSS is a simple, fast and easy‐to‐use awareness tool for cow's milk‐related symptoms [8]. CoMiSS < 10 indicates symptoms possibly related to cow's milk protein intake, but not indicated as cow's milk allergy (CoMiSS ≥ 10) [9]. Goat milk based infant formula (GMF) may be a promising alternative for cow's milk based infant formula (CMF) for infants suffering from GI symptoms with CoMiSS < 6.

Only limited research has been done to this date on GMF‐consumption and GI issues in infants. A case‐series study (*n* = 20) examined the potential influence of GMF on symptoms of constipation in infants that were fed CMF before the introduction of GMF for 3 weeks. The authors found significantly softer stools (Bristol stool score 2 vs. 3, *p* < 0.05) and less crying by the infants (3 h/d vs. 1 h/d; *p* < 0.001) [10]. A prospective cohort study of 12 months by Han et al. concluded that stool consistency of infants fed CMF was harder than of GMF‐fed and breastfed infants (*n* = 976). Furthermore, GMF fed infants' bowel motions tended to be similar to those of breastfed infants ( > 7/d; *p* < 0.05) while CMF‐fed infants had fewer bowel motions (mostly 1–2/day) compared to breast‐fed infants [11]. A randomized controlled trial (RCT) by Grant et al. showed that median daily bowel movement frequency was higher for GMF‐fed infants in comparison to CMF fed infants (2.4 vs 1.7, *p* = 0.01) [12]. In a case study (*n* = 3), the consumption of GMF for 3 weeks was well tolerated in 11‐month‐old infants who experienced symptoms related to cow's milk consumption, and CoMiSS reduced after the introduction of GMF [13].

Due to the limited research, the potential impact of GMF consumption on GI symptoms in infants needs to be further investigated. Therefore, a cross‐sectional online‐survey was designed aiming to study the occurrence of GI symptoms and the possible role of GMF to relief GI symptoms.

## 2 Methods

### 2.1 Study Design and Population

A cross‐sectional observational online anonymous survey on GI symptoms in infants aged 0–24 months fed GMF has been developed and conducted in Brazil, Mexico, Russia, and The Netherlands. The GMF of interest was commercially available Kabrita stage 1 or 2 (Ausnutria, B.V., the Netherlands). Participants who had purchased Kabrita were asked to complete a voluntary survey regarding their experience and observation on their infant's GI complaints. No inclusion criteria were set at the time point of sending out the survey. The survey consisted of 30 questions and was online from June 2021 until November 2022. The study was registered with the US Library of Medicine (clinicaltrials. gov) with identifier NCT06755424.

### 2.2 Human Ethics and Consent to Participate Declarations

The survey and associated data collection do not meet the criteria for human subject research and therefore approval by an institutional and/or licensing committee is not needed, and participant consent is not required. Participation in the survey was anonymized and completely voluntary.

### 2.3 Study Outcomes

The primary outcome of this study were GI symptoms as assessed by the Cow's Milk‐Related Symptom Score (CoMiSS) after introduction of GMF. The CoMiSS was developed by an expert panel to increase the awareness of health care professionals for the presence and severity of symptoms, which might be related to cow's milk protein intake [8]. The CoMiSS is a validated and easy‐to‐use clinical tool with an estimated sensitivity between 20% and 77% and a specificity of 54%–92% to suspected cow's milk allergy [9]. The CoMiSS assesses the stool pattern using the Bristol Scale (hard stools, normal stools, soft stools, liquid stools, watery stools); skin symptoms of atopic eczema on head, neck and/or trunk as well as on arms, hands and/or feet; skin symptoms of urticaria; respiratory symptoms, regurgitation; and crying without any obvious cause in hours per day. The total score ranges from 0 to 33, where lower scores indicate less severe symptoms [7]. The exact sub‐categories of the CoMiSS per symptom can be found in Table 3. As a secondary study outcome, the survey inquired retrospectively about GI symptoms before introduction of Kabrita and whether these symptoms improved minimally 14 days after introduction. These symptoms included gassiness (specified as burping, passing gas, bloating, abdominal pain and/or colic); skin symptoms (specified as rashes, and/or presence of eczema); watery stools or diarrhea; hard stools or constipation; and crying without any obvious cause.

### 2.4 Exposure

Kabrita fulfils the national requirements for infant formulas. The exact compositions of Kabrita differ slightly between countries, according to national criteria. The kcal content per 100 ml (13.2–13.5 g powder + 90 ml water) ranges from 66 to 68 kcal, protein from 1.3 to 1.7 g, carbohydrates from 7.3 to 8.2 g, and total fat from 3.4 to 3.5 g. Vitamin and mineral contents are similar among countries.

The main exposure of interest was the proportion of fed GMF of the infants' total feed (exclusively 100%, predominantly > 60%, often 10%–60%, occasionally < 10%). Additionally, the infants' consumption of GMF per day in ml was monitored. The amount was calculated by multiplying the consumed number of bottles per day by the consumed volume per bottle.

### 2.5 Covariates

Infant characteristics, that is, age in months, sex (girl, boy, unknown), birthweight ( < 2500 g, 2500–4500 g, > 4500 g), being term (gestational age 37–42 weeks), country of residence (Brazil, Mexico, Russia, The Netherlands), previous feeding regime (CMF, another GMF than Kabrita, a formula based on soy, breastmilk or “other”), the infants age when Kabrita was introduced, parent's perception of a generally healthy status of their infant (y/n), use of any medication for functional GI disorders (y/n), and household smoking (inside the house, outside the house, no smoking) were assessed by the survey.

### 2.6 Statistical Analyses

Exclusion criteria for the study population were age > 24 months and taking medication for functional GI disorders. Of the infants meeting the inclusion criteria, the infants regularly consuming Kabrita ( > 10%) for > 14 consecutive days were assigned to the GMF‐consumer group. The infants who were only occasionally or not fed with Kabrita ( < 10%) or fed < 14 consecutive days with Kabrita were included in the GMF‐non‐consumers group, and analyses were done to compare the CoMiSS of the GMF‐consumers with the CoMiSS of the GMF‐non‐consumers. Tests for normality (QQ plots) were performed. Descriptives were performed to present the characteristics among GMF‐consumers and GMF‐non‐consumers as medians with interquartile range (IQR) for skewed variables and *n* (%) for categorical variables.

Differences of the CoMiSS (dependent variable) between sex and country of residence categories (independent variables) were explored using the Independent‐Samples Median Test to estimate potential differences in median CoMiSS. To test for differences in median CoMiSS across age, Poisson regression was performed while correcting for smoking in household. To compare overall median CoMiSS as well as per sub‐categories between GMF‐consumers versus GMF‐non‐consumers, Poisson regression while correcting for smoking in household, age, sex, and country of residence, and Chi‐Square Test for dichotomous variables were performed. The data was analyzed using IBM SPSS Statistics 29. A *p*‐value of < 0.05 was considered statistically significant.

## 3 Results

### 3.1 Study Population's Characteristics

**Figure 1:**
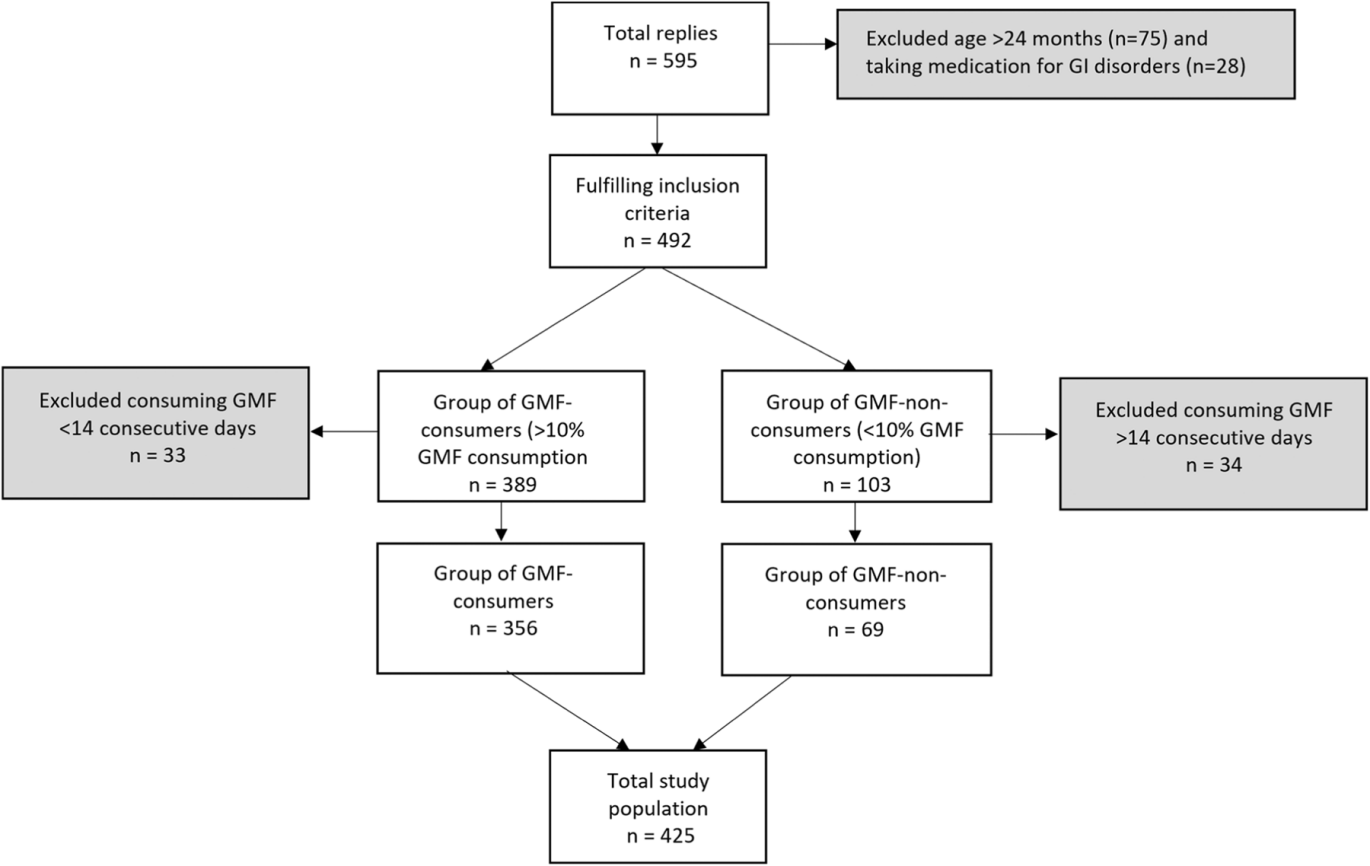
Flowchart of participants.

In total, 595 responses were collected (146 in Brazil, 135 in Mexico, 304 in Russia, 10 in The Netherlands), of which 425 were included for analysis (Figure 1). The study population of GMF‐consumers mostly consisted of 0–6 months‐old infants (61%) with a median age of 5 months (Table 1). Sex was almost equally distributed (44% girls, 53% boys, 3% unknown). Half of the GMF‐consumers resided in Russia, 28% in Mexico, 20% in Brazil and 2% in The Netherlands. Most infants had a birth weight between 2500 and 4500 g (91%), were term infants (83%) and were considered “generally healthy” (86%). In most of the infant's households nobody smoked (69%).

**Table 1:** Characteristics of GMF‐consumers (*n* = 356) and GMF‐non‐consumers (*n*–69).

		GMF‐consumers	GMF‐non‐consumers
		*n* (%) or mean (SD)	*n* (%) or mean (SD)
Age	0*–*6 months	217 (61)	36 (52)
	7*–*12 months	82 (23)	21 (30)
	13–24 months	57 (16)	12 (17)
Sex	Female	156 (44)	34 (49)
	Male	190 (53)	35 (51)
	Unknown	10 (3)	0 (0)
Country of residence	Brazil	72 (20)	19 (28)
	Mexico	101 (28)	4 (6)
	Russia	177 (50)	46 (67)
	The Netherlands	6 (2)	0 (0)
Birthweight	< 2500 g	24 (7)	4 (6)
	2500–4500 g	324 (91)	65 (94)
	> 4500 g	8 (2)	0 (0)
Term infant		295 (83)	59 (86)
Perceived as generally healthy		307 (86)	48 (70)
Someone smoking in household	Inside the house	21 (6)	5 (7)
	Outside the house	90 (25)	21 (30)
	No	245 (69)	43 (63)
Proportion of GMF of total food	Exclusively (100%)	206 (58)	—
	Predominantly (> 60%)	78 (22)	—
	Often (10%–60%)	72 (22)	—
	Occasionally (< 10%)	—	22 (32)
	No (0%)	—	47 (68)
Consumption of GMF/d (ml)		675 (SD 357)	—
Previous feeding regime	Human milk	190 (53)	—
	CMF	115 (32)	—
	GMF other than Kabrita	16 (5)	—
	Soy‐based formula	6 (2)	—
	Other	29 (8)	—
Age when switched to Kabrita	Between 0–6 months	291 (82)	—
	Between 6–12 months	50 (14)	—
	> 12 months	15 (4)	—
CoMiSS	Median	1	2
	IQR	4	6
	Range	0–14	0–13
	Score < 6	317 (89)	54 (78)

The majority of GMF‐consumers was fed “exclusively with GMF” (58%), 22% “predominantly” and 20% “often.” The mean (SD) consumption of GMF per day was 675 ml (357.28). Most GMF‐consumers were 0‐6 months old (82%) when GMF was introduced to their diet and were fed human milk before (53.2%), a third of the GMF‐consumers (32.4%) switched from CMF to Kabrita, 4.5% from another GMF than Kabrita, 1.7% from a soy‐based formula and 8.2% switched from “other” to Kabrita.

### 3.2 GI Symptoms Before and After Introduction of Kabrita

**Figure 2:**
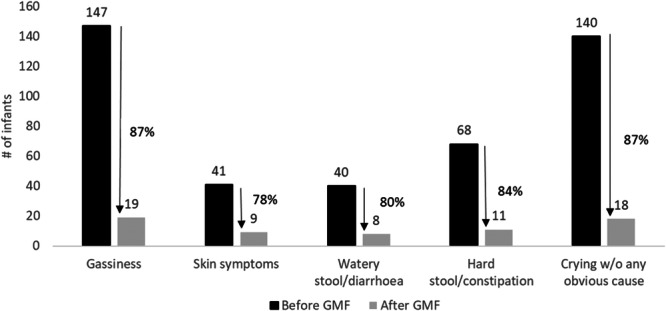
Number of infants with symptoms before and after introduction of GMF among GMF‐consumers (*n* = 356).

Among GMF‐consumers, the most common reported complaint in infants before the introduction of GMF was gassiness (41%), followed by crying without any obvious cause (39%), and hard stools or constipation (19%). Skin symptoms (12%), and watery stools or diarrhea (11%) were the least often reported symptoms before introduction. Most parents (76%) reported that their infant experienced, in general, fewer complaints after introduction of GMF. For the specific GI symptoms, 87% of infants who experienced gassiness before the introduction of GMF, did not report gassiness after introduction. For skin symptoms, 78% lower frequency of reporting skin symptoms after introduction, for watery stools or diarrhea 80%, and for hard stools or constipation 84% after introduction. Crying without any obvious cause was lower after introduction of GMF for 87% of infants (Figure 2).

The highest prevalence of minor complaints before introduction of Kabrita was reported in former CMF‐fed infants (87%), followed by other GMF (56%), soy‐based formula (50%), and “other” (45%). Former breastfed infants showed the lowest prevalence (44%) of minor complaints in general before introduction of GMF (Table 2).

**Table 2:** Previous feeding regime and prevalence of GI symptoms before introduction of Kabrita (*n* = 356).

Previous feeding regime	Minor complaints in general	Gassiness	Skin symptoms	Watery stools or diarrhea	Hard stools or constipation	Crying without any obvious cause
Breastfeeding (*n* = 190)	44.2%	56.0%	13.1%	16.7%	22.6%	33.7%
CMF (*n* = 114)	86.8%	64.6%	24.2%	23.2%	37.4%	53.5%
GMF other than Kabrita (*n* = 16)	56.3%	55.6%	0%	0%	44.4%	37.5%
Soy‐based formula (*n* = 6)	50.0%	33.3%	0%	0%	33.3%	16.7%
Other (*n* = 29)	44.8%	81.3%	18.8%	12.5%	25.0%	24.1%

### 3.3 CoMISS Among Study Population

The overall CoMiSS among GMF‐consumers was low (median 1.00, IQR 4.00, range 0–14) (Table 1). Almost all GMF‐consumers showed a CoMiSS ≤ 6 (*n* = 317, 89%), indicating symptoms commonly present in healthy infants.

The GMF‐non‐consumers showed a higher CoMiSS (median 2.00, IQR 6.00, range 0–13) and 78% (*n* = 54) had a CoMiSS ≤ 6 than the GMF‐consumers (Table 1). Poisson regression showed that GMF‐consumers had a 0.52 points significantly lower CoMiSS than GMF‐non‐consumers while corrected for smoking in household, age, sex, and country (*p* = < 0.001) (data not shown).

The subscore crying without any obvious cause was significantly lower for GMF‐consumers compared to GMF‐non‐consumers (*p* = 0.003) as well as for stool consistency (*p* = < 0.001), and urticaria (median test *p* = 0.001). The remaining categories (regurgitation, eczema, respiratory symptoms) did not show significant differences in CoMiSS between GMF‐consumers and GMF‐non‐consumers (Table 3).

**Table 3:** Median (IQR, gray lines) or *n* (%) per symptom contributing to the CoMiSS among GMF‐consumers (*n* = 356) and GMF‐non‐consumers (*n* = 69).

	GMF‐consumers	GMF‐non‐consumers	*p*‐value
Overall CoMiSS	1.00 (4.00)	2.00 (6.00)	< 0.001
Crying without any obvious cause	0.00 (0.00)	0.00 (0.00)	0.003
< 1 h/d	301 (85)	55 (80)	
1–1.5 h/d	36 (10)	7 (10)	
1.5–2 h/d	11 (3)	3 (4)	
2–3 h/day	3 (1)	1 (1)	
3–4 h/d	2 (1)	1 (1)	
4–5 h/d	0 (0)	1 (1)	
> 5 h/d	3 (1)	1 (1)	
Regurgitation	0.00 (0.00)	0.00 (0.00)	0.72
0–2 episodes/d	286 (80)	57 (83)	
3–5 small volume	43 (12)	5 (7)	
> 5 episodes of > 1 coffee spoon	17 (5)	3 (4)	
> 5 episodes of + half of the feed in < half of the feeds	1 (0)	2 (3)	
Continuous regurgitation of small volumes > 30 min after each feed	8 (2)	2 (3)	
Regurgitation of the complete feed after each feeding	1 (0)	0 (0)	
Stool consistency	0.00 (0.00)	0.00 (2.00)	< 0.001
Hard stool (type I)	27 (8)	9 (13)	
Normal stools (type 2 or type 3)	286 (80)	57 (83)	
Soft stools (type 4)	29 (8)	10 (15)	
Liquid stools (type 5A)	12 (3)	7 (10)	
Watery stools (type 5B)	2 (1)	0 (0)	
Urticaria	0.00 (0.00)	0.00 (3.00)	0.001*
Present	37 (10)	17 (25)	
Absent	319 (90)	52 (75)	
Eczema	0.00 (0.00)	0.00 (0.00)	0.19
Absent (less than 1/3 of head, neck and/or trunk)	318 (89)	66 (96)	
Mild (1/3–2/3 of head, neck and/or trunk)	30 (8)	1 (1)	
Moderate (over 2/3 of head, neck and/or trunk)	6 (2)	2 (3)	
Severe (head, neck and/or trunk fully covered with eczema)	2 (1)	0 (0)	
Respiratory symptoms	0.00 (0.00)	0.00 (0.00)	0.31
Absent	320 (90)	60 (87)	
Mild	30 (8)	8 (12)	
Moderate	6 (2)	1 (1)	

* Chi‐Square test for dichotomous variable was performed, *p*‐values adjusted for smoking in household, age, sex, and country of residence.

## 4 Discussion

The results of our study on parent‐reported GI symptoms in infants consuming GMF showed an overall low CoMiSS, indicating severity of symptoms commonly present in healthy infants. For most infants experiencing GI symptoms before introducing GMF, the severity of symptoms was lower after introduction. Analyses demonstrated that the CoMiSS was significantly lower, meaning fewer symptoms, in GMF‐consumers compared to GMF‐non‐consumers.

Our findings in infants consuming GMF showing an overall low score on crying in hours per day (85% cried < 1 h/d) as well as a high proportion of infants with normal stools (80%), are similar to the findings of Infante et al. (2018). This study showed improvement of GI symptoms in infants after introducing GMF for 3 weeks: better stool consistency (from harder to softer stool) and less crying (from 3 h/24 h to 1 h/24 h) [10]. Han et al. showed a similar finding by concluding that CMF fed infants had harder stools and less frequent bowel motions in than GMF fed infants [11]. Moreover, our finding on lower GI symptoms after the introduction of GMF is in alignment with the study by Salsberg et al., who found a reduction in cow‐milk related symptoms after the introduction of GMF [13]. Due to our study design, drawing conclusions about causality between GMF‐consumption and GI symptoms is not possible. Nevertheless, the results might suggest that there is a benefit of GMF‐consumption in healthy infants.

Literature on suitability of GMF for infants show adequate growth, acceptable GI tolerance and safety outcomes. GI tolerability was assessed either by mean number of stool motions/day [14], frequency of adverse events such as vomiting, diarrhea, constipation or food refusal [12] or risk of an adverse health condition, including GI illness and reflux [15]. None of these studies showed a differences between GMF and CMF in GI tolerability. In a case series study in infants (*n* = 20) an improvement of stool characteristics and reduction in crying was seen, however, GI symptoms were not scored in this study [10]. Our survey results does show a significant lower CoMiSS in GMF‐consumers when compared to GMF‐non‐consumers. Future randomized controlled studies should investigate whether CoMiSS might be a better tool to assess GI tolerability in infants consuming GMF and CMF.

Due to the different compositions of Kabrita between countries, it would have been possible that the CoMiSS was different between countries. The prebiotics (GOS and FOS), for example, not present in Kabrita Brazil, are known for their effect on softer stools and an increased stool frequency [16]. *Bifidobacterium animalis subsp. lactis*, BB‐12, only present in Kabrita Russia, has been associated with less crying in infants [17]. However, no significant difference in the CoMiSS between countries (*p* = 0.45) was observed, which might be due to the use of questionnaires not being sensitive enough to detect these differences and should be studied in randomized controlled studies.

### 4.1 Potential Mechanisms

The biological mechanism through which GMF might have a beneficial effect on GI symptoms could be its protein digestion. It has been previously shown that the initial protein digestion of GMF is faster than the digestion of CMF, while their protein quality is similar [18]. In comparison to CMF, GMF forms under gastric conditions smaller flocs of aggregated protein and smaller oil droplets. These may explain the faster gastric emptying since smaller and softer curds can promote digestive activity of the gastric proteases, while larger particles will not easily pass the gastric tract since they need to be broken down by pepsin before emptying is possible [19, 20]. The coagulation and the size of oil droplets may depend on the protein composition, which differs between GM, CM and human milk (HM). In contrast to CM, GM shows a lower casein content and a different protein profile/composition. GM contains less α‐s1‐casein, has a larger β‐casein proportion and a larger casein micelle size than CM [19]. In HM, β‐casein is the major protein while α‐s1‐casein is not present. The digestion kinetics of GMF are more comparable to HM than those of CMF [20], which is to consider positively in view of HM being the gold standard in infant nutrition [21]. However, the clinical relevance of the faster digestion of GMF compared to CMF on GI symptoms in infants is yet ambiguous. Previous research only refers to the protein physicochemical properties and kinetics of GM and not of GMF and its digestion and influence on GI symptoms in infants.

### 4.2 Limitations

The present study has the following limitations. The research was done using a cross‐sectional survey design, where data was collected at a single time‐point, which does not allow conclusions about causality. Since this was an observational study, the survey has been sent out to parents who were already been purchasing Kabrita. Although we were able to correct for the confounding factors age, sex, country of residence and smoking, other possible confounding factors such as education and socioeconomic status were not recorded but could have an impact the results obtained here [22]. Including these factors in analysis might have led to higher quality of effect estimates and less residual confounding. Furthermore, measurement errors may have occurred due to subjective answers, as the survey was distributed among parents already feeding Kabrita to their child. Therefore, most participants' perception of Kabrita might have been positive because they believe it is beneficial or already had positive experiences, which could have contributed to the positive results regarding GMF consumption. The retrospective observational set‐up might have led to recall bias (participants had to answer the questions about GI symptoms before introduction of Kabrita retrospectively).

### 4.3 Strengths

The present study contains the following strengths. This study is the first online survey focused on GMF introduction, performed in four countries representing Europe and South‐America providing real‐world evidence. Furthermore, the CoMiSS used in this study is a validated tool. Vandenplas et al. concluded that the use of the CoMiSS by parents without additional training to assess GI symptoms of their infant results in a reliable score [23].

### 4.4 Conclusion and Future Studies

In conclusion, this study of healthy infants showed a low prevalence of GI symptoms and parents reported lower prevalence of GI symptoms after introduction of GMF. CoMiSS in GMF‐consumers was significantly lower when compared to consumers of other infant formulas. This real‐world‐evidence research gives indication that GMF might be a valuable alternative for infants suffering from mild GI complaints. This could help healthcare professionals such as pediatricians, pediatric nurses and midwifes to recommend GMF such as Kabrita in infants with mild GI symptoms when breastfeeding is not possible. A follow‐up, well‐powered, double‐blinded study with a standardized CMF control product is needed to confirm the beneficial outcomes in GI symptoms when using GMF.

## Author Contributions

**Karen Knipping:** formal analysis, investigation, methodology. **Juliane Böhme:** data curation, formal analysis, investigation, methodology. **Dominique Goossens:** conceptualization, formal analysis, project administration. **Linde Lee:** conceptualization, formal analysis, project administration. **Lucie Zee:** conceptualization, formal analysis, project administration.

## Conflicts of Interest

The study was funded by Ausnutria B.V. Karen Knipping, Dominique Goossens, Linde van Lee and Lucie van der Zee are employee of Ausnutria B.B and were involved in study design; collection, analysis, and interpretation of data; writing of the report; and the decision to submit the report for publication. Juliane Böhme declare no conflict if interest.

### Transparency statement

The corresponding Karen Knipping affirms that this manuscript is an honest, accurate, and transparent account of the study being reported; that no important aspects of the study have been omitted; and that any discrepancies from the study as planned (and, if relevant, registered) have been explained.

## Data Availability

The data that support the findings of this study are available from the corresponding author upon reasonable request.
